# Body Fat and Body-Mass Index among a Multiethnic Sample of College-Age Men and Women

**DOI:** 10.1155/2013/790654

**Published:** 2013-04-08

**Authors:** Catherine L. Carpenter, Eric Yan, Steve Chen, Kurt Hong, Adam Arechiga, Woo S. Kim, Max Deng, Zhaoping Li, David Heber

**Affiliations:** Center for Human Nutrition, David Geffen School of Medicine at UCLA, 14-193 Warren Hall, 900 Veteran Avenue, P.O. Box 951742, Los Angeles, CA 90095, USA

## Abstract

Obesity prevalence and average body composition vary by US race and gender. Asian Americans have the lowest prevalence of obesity. Relying on body-mass index (BMI) to estimate obesity prevalence may misclassify subgroups that appear normally weighted but have excess body fat. We evaluated percentage body fat (PBF) and BMI to determine whether BMI reflects PBF consistently across different races. 940 college students were recruited from a local public university over four consecutive years. We measured PBF by bioelectrical impedance analysis (BIA), weight by physicians' scales, and height with stadiometers. Our sample comprised Asians (49%), Caucasians (23%), Hispanics (7%), and Other (21%). Participants averaged 21.4 years old; BMI was 22.9 kg/m^2^; PBF was 24.8%. BMI and PBF varied significantly by race and gender (*P* value = 0.002 and 0.005 for men; 0.0009 and 0.0008 for women). Asian-American women had the lowest BMI (21.5 kg/m^2^) but the second highest PBF (27.8%). Linear association between BMI and PBF was the weakest (*r* ^2^ = 0.09) among Asian-American women and BMI had the poorest sensitivity (37%) to detect PBF. The high PBF with low BMI pattern exhibited by Asian-American women suggests that they could escape detection for obesity-related disease if BMI is the sole measure that estimates body composition.

## 1. Introduction

Body-mass index (BMI), an important indicator of obesity prevalence in large populations, generally reflects degree of fatness among individuals. Body-mass index can however over- or underestimate adiposity depending upon certain circumstances.

Accurate determination of obesity has become exceedingly important because of major health threats posed by excess adiposity. Obesity is associated with increased incidence of cardiovascular disease, diabetes, sleep apnea, degenerative joint disease, and site-specific cancers [1–6]. Moreover, high obesity prevalence could potentially result in shortened life expectancy in the coming years [7] and excess mortality [8, 9]. Because of the present and future health problems associated with excess adipose tissue, underestimation of obesity, particularly in young adults who might otherwise appear to have normal BMI measures, could lead to false conclusions about body composition and future health status. Underestimation of body fatness in young women for instance may be problematic for future risk of diseases such as breast cancer. Patterns of excess adipose tissue established early in adulthood could promote the occurrence of obesity at menopause, a known risk factor for breast cancer [10–12].

Validation studies have evaluated accuracy of BMI in estimating body fatness, by comparison to more refined measures such as bioelectric impedance analysis (BIA) and dual energy absorptiometry (DXA) [13–21]. Some studies observed low sensitivity of BMI to detect obesity in general [14, 20], while others concluded that BMI was most inaccurate in detecting obesity among intermediate ranges of BMI [13, 17, 20, 21]. In addition accuracy of BMI to detect body fatness appears to be affected by ethnicity [15, 18–20], gender [17, 21], and age [13, 19–21].

The prevalence of inactivity has increased among all age groups and is thought to be a major contributor to the obesity epidemic [22]. With the rise in sedentary behavior, potentially greater numbers of young and middle aged adults may be susceptible to accumulation of unhealthy amounts of adipose tissue without significant weight change. We undertook a study of young adult college age adults to examine the relationships between percentage body fat and body-mass index among a multiethnic sample living in Southern California.

## 2. Materials and Methods

### 2.1. Study Population

Serial cross-sectional samples of college-age men and women were recruited during winter quarter of four consecutive years, 2006, 2007, 2008, and 2009 from an undergraduate physiology course at a major public university in Southern California. All 1029 students in the course over the three years were eligible and subsequently enrolled (241 in 2006, 242 in 2007, 239 in 2008, and 307 in 2009) into the study. Data from 940 students with complete covariate information were included.

### 2.2. Human Research

The study was approved by the Institutional Review Board of the University of California at Los Angeles. We certify that all applicable institutional and governmental regulations concerning the ethical use of human volunteers were followed during this research.

### 2.3. Demographic Variables

Ethnicity and racial background were self-identified using the following categories: White/Caucasian; Hispanic; Black/African American; Middle Eastern; Indian; Native American; Asian (Eastern); Asian (Southeast); Pacific Islander; and Other. A blank space to write in “Other” was provided, and, additionally, multiple categories could be marked in the event of mixed race or ethnicity. Age was self-reported.

Because of small numbers of individuals in some of the racial/ethnic groups and to increase the power of our stratified analyses we combined race/ethnicity into four groups: Asian, White, Hispanic, and Other. Eastern and Southeastern Asian groups were combined into Asian. Other category consisted of multiple ethnicities, recorded Other, Middle Eastern, Native American, African American, and Pacific Islanders. Self-reported White/Caucasian and Hispanic were retained as singular categories.

### 2.4. Anthropometric Measurements

Trained clinical technicians conducted all anthropometric measurements. Subjects were weighed while wearing no shoes. Body weight was measured with a physicians' scale. Heights were taken with a stadiometer (Detecto-Medic; Detecto-Scales; Brooklyn, NY).

Bioelectrical impedance analysis (BIA), used to estimate percent body fat (PBF) and fat and fat free mass, was conducted with a quadripolar BIA device (310e Bioimpedance analyzer; Biodynamics, Inc., Seattle, WA). Fat-free mass and fat mass are estimated with a regression equation based on data obtained through comparison with bioimpedance estimates with hydrodensitometry (Biodynamics, Inc., Seattle, WA). The equation used by the present study, and also utilized in our earlier study [13], estimates FFM = (a × Ht^2^) + (b × Wt) + (c × A) + (d × *R*) + e, where FFM is fat-free mass, Ht is height (cm), Wt is weight (kg), A is age (years), and *R* is impedance (Ω). The constants, a through e, are proprietary information of Biodynamics, Inc. We utilized the model for estimation of FFM for our entire study population. We constructed categories of body-mass index using the WHO International Criteria for all populations (<18.5 kg/m^2^; 18.5–24.9 kg/m^2^; 25–29.9 kg/m^2^; >30 kg/m^2^) [23], and the WHO criteria for Asian populations with suggested public health action (<23 kg/m^2^; 24–27.5 kg/m^2^; 27.6–32.49 kg/m^2^; ≥32.5 kg/m^2^) [24]. Since there are no accepted cutpoints for percentage body fat [25], we utilized the PBF cutpoints defined in Okorodudu et al., 2010, a diagnostic performance meta-analysis of BMI in relationship with percentage of body fat [26].

### 2.5. Statistical Analysis

Analysis of variance was used to evaluate differences in anthropometric variable means according to race and gender. We further analyzed associations between BMI and PBF by constructing multiple linear regression models adjusting for gender and ethnicity and linear regression models of BMI and PBF according to whether Asian or Caucasian separately for males and females. We plotted scatter distributions of BMI and PBF for Asian and Caucasian males and females.

To provide estimates of sensitivity and specificity of BMI to predict PBF, we evaluated distributions of gender and ethnic subgroups according to BMI and PBF. If we consider PBF measured by BIA as the gold standard, we computed sensitivity as the proportion of participants classified as obese by BMI (≥30.0 kg/m^2^) and PBF (≥25.0% for men and ≥30.0% for women) divided by the total classified as obese by PBF. We computed specificity as the proportion of participants classified as nonobese (normal or overweight) by BMI (<30.0 kg/m^2^) and nonobese (normal or overweight) by PBF (<25.0% for men and <30% for women) divided by the total classified as nonobese by PBF [27]. We restricted our estimation of sensitivity and specificity to Asian-Americans and Whites because the other ethnic groups had insufficient numbers to provide stable estimates.

All data analyses were performed using the Statistical Analysis System Version 9.2 (Statistical Analysis System 2008, Cary, NC, USA). All reported *P* values assume a two-sided alternative hypothesis. *P* values less than or equal to 0.05 were considered significant.

## 3. Results

Most study participants (see Table 1) were Asian-American (49%), with 23% White, 7% Hispanic, and 21% Other. Most were females (60%). Age distribution was fairly narrow, with subjects averaging 21 years old (standard deviation was 1.6 years). Average body-mass index (BMI) was 23 kg/m^2^, weight 65.4 kg (145 pounds), and height 169 centimeters (cm) (66 inches). Total PBF estimated by BIA was 25%. Fat mass averaged 17 kilograms (kg); fat-free mass 49 kg.

Almost all anthropometric measures were significantly different according to race and according to gender and race (see Table 2). Hispanics and men of other ethnicities had the highest BMI, both averaging 26 kg/m^2^ and 25 kg/m^2^, respectively, while Hispanic females (mean = 30%), Asian females (mean = 28%), and Other females (mean = 29%) had the highest percentage body fat. Asian-American males (mean = 174 cm) (68 inches) and Asian-American females (mean = 161 cm) (63 inches) were the shortest among the racial/ethnic subgroups.

**Figure 1:**
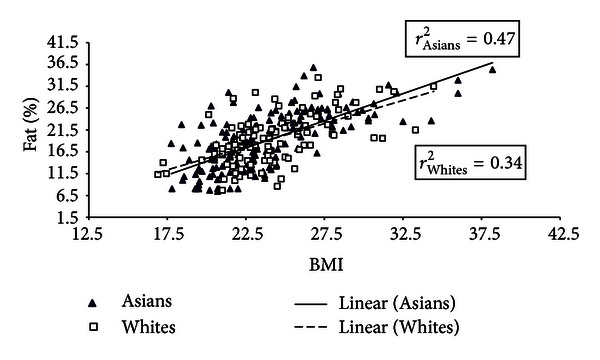
Distribution of body-mass index and percentage body fat for males according to race/ethnicity.

**Figure 2:**
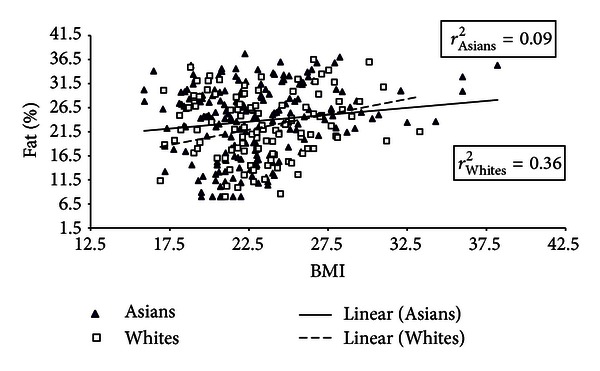
Distribution of body-mass index and percentage body fat for females according to race/ethnicity.

Results from multiple linear regression analyses suggested that 52% of the variability (*r* ^2^ = 0.52) in PBF was explained by BMI, ethnicity, and sex. We further compared degree of association between BMI and PBF for both Asian-Americans and Whites by constructing separate scatter plots for males and females (see Figures 1 and 2). In addition we computed fit of association between BMI and PBF using linear regression models. Among men, the association between BMI and percent body fat was fairly strong and linear for Asian-Americans (*r* ^2^ = 0.47) and less precisely associated for Whites (*r* ^2^ = 0.34). Among females however the association was weaker, particularly among Asian-Americans (*r* ^2^ = 0.09).

We utilized the WHO International (BMI ≥ 30.0 kg/m^2^) and the WHO Asian (BMI ≥ 27.5) cutpoints for obesity [23, 24] and subclassified according to PBF cutpoints for men (≥25%) and women (≥30%) defined in Okorodudu et al. [26] to estimate frequency of individuals who were correctly classified by BMI and individuals who were not (see Table 3). If we consider PBF as a more accurate estimation of obesity, the sensitivity of BMI to predict PBF in Asian-American men was 91%. Among Asian-American women however, the sensitivity was much lower at 37% (see Table 3). Specificity in Asian-American women was higher at 81%, while the specificity of BMI to predict nonobese PBF was poorer in Asian-American men (63%). Whites showed a similar pattern to Asian-Americans, although the differences were less striking. The sensitivity of BMI to predict PBF among White men (70%) was higher than White women (50%), while the reverse was true for specificity. The specificity of BMI to predict non-obese PBF was higher among White women (98%) compared to White men (72%).

## 4. Discussion

Our study was designed to evaluate relationships between percentage body fat and body-mass index among a multiethnic sample of college-age men and women living in Southern California. We were interested in determining the extent to which excess adiposity might be occurring among normal to intermediate ranges of body-mass index and whether these relationships may vary according to gender and ethnicity. We utilized comparative measures between body-mass index and percentage body fat to characterize instances where low degree of association may represent elevated body fat in the context of normal BMI measurements.

We studied college-age young adults because this age group, in particular, may be more likely to have BMI measurements in the normal to intermediate range. The average body-mass index for US men (27 kg/m^2^) and women (26.5 kg/m^2^) between the ages of 20 are 29 is the lowest of all adult age groups younger than age 80 years [28]. The average BMI in our study population was similar to US population norms. Average BMI for males in our study was 24.4 kg/m^2^ and females 22.0 kg/m^2^.

Estimates for body composition among the college-aged sample were strikingly different for gender and race. Asian men (23.7 kg/m^2^) and women (21.5 kg/m^2^) had the lowest mean BMI among the study sample, while Hispanic men (25.9 kg/m^2^) and women (23.5 kg/m^2^) had the highest mean BMI. Percentage body fat did not follow the same distribution pattern however. While Asian women had the lowest BMI, they did not have the lowest percentage body fat. Asian women had 27.8% body fat, while Caucasian women, lower than Asian women, had 26.9%. Hispanic women had the highest percentage body fat (29.8%).

The correlation between BMI and PBF for the total sample, while moderate, did not indicate variation according to gender and race subgroups. We computed correlation coefficients between BMI and PBF and found that the overall partial correlation between BMI and percentage body fat in our study population, adjusting for race and gender, was 0.63. Partial correlation for men was 0.63 and women 0.46, both adjusting for race (data not shown). Our correlation for men was similar to a study using the Third NHANES sample [21]. Their study reported a correlation of 0.69 among men in the 20 to 29 age group [21]. Our reported correlation for women (0.46), on the other hand, was much lower than that reported in NHANES (0.89) [21].

Age, gender, and ethnicity have been found in several studies to affect strength of relationship between BMI and percentage body fat [13, 15, 17–21]. In the population-based NHANES III study, correlations became weaker as age increased [21]. A study of body fatness among 706 African Americans and Caucasian men and women in New York City found that older subjects had higher percentage body fat with similar BMI measurements compared to younger subjects from both racial and gender subgroups [29]. In a recent multiethnic population survey from NHANES 1999–2004 of BMI and other anthropometric measures, agreement of BMI with percentage body fat varied significantly by race-ethnicity categories [25]. The present study population consisted of young college-age adults with mean and median ages of 21.5 and 21.0, respectively, suggesting that based on previous studies, we ought to be observing stronger agreement between BMI and percentage body fat.

Gender also affects the degree to which BMI predicts body fat [13, 17, 21, 25, 29]. Females have higher percentages of body fat compared to males of all ages and ethnic groups [21, 25], and, for an equivalent BMI, women have significantly greater amount of total body fat than men throughout the entire adult life span [21]. Among all four ethnic subgroups in the present study, females averaged a higher percentage body fat, but lower BMI than males. In all ethnic groups except Whites, females had weaker associations between percentage body fat and BMI than males.

The relationship between PBF and BMI has been shown to differ according to ethnic origin [30]. A meta-analysis concluded that for the same PBF, African Americans and Polynesians have higher BMI compared to Caucasians. In contrast, Chinese, Ethiopians, and Thai BMI measurements are lower than Caucasians [31]. Other studies of Asians have shown that Taiwanese subjects had a relatively lower BMI but higher PBF than Caucasians [18]. Similarly, Indonesians had higher PBF but lower BMI compared to Dutch Caucasians [32], and Japanese young men living in Japan and Australia had greater body fat distribution but lower BMI compared to Australian Caucasians [19]. In our multiethnic sample of young adults, the linear association between BMI and PBF was stronger for Asian men (*r* ^2^ = 0.47) than for Caucasian men (*r* ^2^ = 0.34), while the reverse was true for women. The association between BMI and PBF was the weakest for Asian women (*r* ^2^ = 0.09) compared to Caucasian women (*r* ^2^ = 0.36).

High percentage of body fat occurring at lower BMIs has also been observed among younger Japanese in a multinational study of Japanese, Caucasians, and African Americans conducted in Japan, the United Kingdom, and the United States [33]. The study used DXA, underwater weighing, and BMI, to develop prediction formulas that estimated PBF using a four-compartment model. According to their prediction model, Asians had a significantly higher percentage body fat for any given BMI than Caucasians and African Americans [33].

The low degree of association between BMI and PBF that we observed for young Asian-American women in particular may signal a present and future risk for obesity-related disease. BMI was a poor predictor of PBF in Asian-American women reflected by a low sensitivity (37%). The low sensitivity and weak association suggest that use of BMI to estimate adiposity may be especially inaccurate in Asian-American women. In a comparative study of body composition in Asian and Caucasian young adult females, results showed a similar PBF (31%) for Taiwanese women aged 20 to 29, with a similar BMI (23.7 kg/m^2^) that we observed [18]. In a comparative study of prepubertal children from China and New York City, similar correlation patterns were observed with Chinese girls having the highest PBF and lowest BMI compared to girls of other geographic and racial origins [34]. In a large cross-sectional study of adiposity from a medical practice in Manhattan, BMI misclassified 48% of women when DXA was used to validate BMI [35].

Our reliance on BIA to estimate PBF measurement may have contributed to potential inaccuracies in our data. A validation study of body fat estimation by BIA compared to DXA conducted among multiethnic women showed that underestimation of lean body-mass was affected by whether being Caucasian or African American [15], although their study was conducted among overweight to obese women, and whether the same underestimation would occur in a younger normal weighted population with a different ethnic distribution is unclear. In another validation study conducted among 5 European populations, the bias in BIA measurement compared to DXA was minor, particularly among subjects younger than age 35 [16]. Our PBF estimates measured by BIA for Asian females (27.8%) and Asian males (18.9%) are close to the PBF observed among Taiwanese females (30.6%) and males (22%) between the ages of 20 and 29 measured by the DXA [18].

## 5. Conclusions

In conclusion we observed striking differences in body composition according to gender and ethnicity among a young adult college-age population. While most males and females of different ethnicities had similar associations between PBF and BMI, Asian-American females represented a special subgroup where BMI did not accurately reflect underlying adiposity. The weight and BMI measurements were representative of normal; however the relative high PBF may put Asian-American females at risk for future obesity-related disease.

## Figures and Tables

**Table 1:** Characteristics of the study population.

Variable	Category	N	Percent
	East Asian	364	37.92
	SE Asian	96	10.00
	African American	11	1.15
	Hispanic	68	7.08
Racial composition	Indian	51	5.31
	Middle Eastern	86	8.96
	Pacific Islander	27	2.81
	White	216	22.50
	Other	41	4.27

	Asian, no Pac Islander	475	49.48
Combined racial groups	White	216	22.50
	Hispanic	68	7.08
	Other, Inc Pac Isl, Mixed	201	20.94

Gender	Male	380	39.58
	Female	580	60.42

Body-mass index (WHO-International)*	<18.5	74	7.71
	18.5–24.9	664	69.17
	25.0–29.9	170	17.71
	30.0+	52	5.42

Body mass index (WHO-Asian populations)**	<18.5	74	7.71
	18.5–22.9	481	50.10
	23.0–27.49	296	30.83
	27.5+	109	11.35

Percent body fat	<20.0	230	23.96
	20.0–24.9	227	23.65
	25.0–29.9	291	30.31
	30.0+	212	22.08

Age (years)	Mean		21.40
	S.D.		1.64

Body mass index	Mean		22.95
	S.D.		3.82

Weight (kg)	Mean		65.37
	S.D.		14.74

Height (m)	Mean		1.69
	S.D.		0.09

Total percent body fat	Mean		24.82
	S.D.		6.94

Fat body mass (kg)	Mean		16.52
	S.D.		11.4

Fat-free body mass (kg)	Mean		49.15
	S.D.		11.76

*WHO: see [23].

**WHO: see [24].

S.D.: standard deviation.

**Table 2:** Study population characteristics according to race/ethnicity and according to race/ethnicity and gender.

Characteristic	Asian		White		Hispanic		Other		*P* value
	Mean	S.D.	Mean	S.D.	Mean	S.D.	Mean	S.D.
	Overall study population								

Age (years)	21.16	1.31	21.66	2.14	21.93	2.12	21.52	1.45	0.0001
Height (cm)	165.89	8.78	172.49	8.99	168.48	9.91	168.91	9.53	0.0001
Body weight (kg)	61.79	13.08	69.07	14.16	69.80	17.07	68.36	16.15	0.0001
BMI (kg/m^2^)	22.31	3.37	23.09	3.60	24.32	4.70	23.84	4.40	0.0010
Percent body fat	24.57	6.97	23.67	6.55	27.30	6.73	25.81	7.06	0.0020
Fat mass (kg)	15.17	5.51	16.41	6.4	19.19	7.54	17.57	6.91	0.0600
Fat-free mass (kg)	46.62	10.82	53.31	10.93	50.69	12.59	50.85	12.76	0.0001

	Males								

Age (years)	21.12	1.36	21.95	2.69	22.71	3.00	21.51	1.66	0.0020
Height (cm)	174.22	6.30	179.02	2.81	177.44	7.14	176.53	7.21	0.0001
Body weight (kg)	72.33	13.37	78.04	11.17	82.46	15.49	78.88	13.83	0.0001
BMI (kg/m^2^)	23.74	3.73	24.38	3.23	25.85	4.54	25.36	3.74	0.0020
Percent body fat	18.86	7.73	19.62	5.86	22.74	6.36	21.81	5.95	0.0005
Fat mass (kg)	14.26	7.40	15.74	6.28	19.38	8.37	17.30	6.96	0.0005
Fat-free mass (kg)	58.03	7.49	62.30	7.58	63.30	9.30	61.60	9.37	0.0010

	Females								

Age (years)	21.19	1.29	21.43	1.56	21.50	1.28	21.54	1.26	0.0600
Height (cm)	161.24	6.15	167.36	6.71	163.58	7.49	162.59	5.92	0.0001
Body weight (kg)	55.92	8.41	62.02	12.16	62.90	13.68	59.65	12.32	0.0001
BMI (kg/m^2^)	21.51	2.87	22.07	3.56	23.48	4.62	22.58	4.51	0.0009
Percent body fat	27.75	4.71	26.85	5.17	29.78	5.58	29.12	6.15	0.0008
Fat mass (kg)	15.67	4.11	16.94	6.47	19.09	7.14	17.79	6.90	0.0010
Fat-free mass (kg)	40.26	6.18	45.09	7.26	43.81	7.97	41.96	7.26	0.0001

BMI: body-mass index.

**Table 3:** Classification of obesity for Asian-American and US white college age adults using body-mass index and percentage body fat.

		Classification of						Classification of			
		obesity in US Whites*						obesity in US Asians**			
		BMI						BMI			
		<30.0 kg/m^2^		≥30.0 kg/m^2^				<27.5 kg/m^2^		≥27.5 kg/m^2^	
		*N*	%	*N*	%			*N*	%	*N*	%
Males						Males					
Percentage	<25%	56	71.8	22	28.2	Percentage	<25%	85	63.0	50	37.0
Body fat	≥25%	5	29.4	12	70.6	Body fat	≥25%	3	8.6	32	91.4
Sensitivity	70.6%					Sensitivity	91.0%				
Specificity	71.8%					Specificity	63.0%				
Females						Females					
Percentage	<30%	89	97.8	2	2.2	Percentage	<30%	172	81.1	40	18.9
Body fat	≥30%	15	50.0	15	50.0	Body fat	≥30%	59	63.4	34	36.6
Sensitivity	50.0%					Sensitivity	36.6%				
Specificity	97.8%					Specificity	81.1%				

*WHO: see [23].

**WHO: see [24].

BMI: body-mass index.

## Abbreviations

PBF: Percent body fat
BIA: Bioelectric impedance analyst
DXA: Dual energy absorptiometry.
